# A Patient Decision Aid App for Patients With Chronic Kidney Disease: Questionnaire Study

**DOI:** 10.2196/13786

**Published:** 2019-11-21

**Authors:** Signe Bülow Therkildsen, Linda Houlind Hansen, Laura Emilie Dinesen Jensen, Jeanette Finderup

**Affiliations:** 1 Department of Orthopedics Oslo University Hospital Oslo Norway; 2 Department of Nephrology Odense University Hospital Odense Denmark; 3 Centre for Eating Disorders Aarhus University Hospital Aarhus Denmark; 4 Department of Renal Medicine Aarhus University Hospital Aarhus Denmark; 5 Department of Clinical Medicine Aarhus University Aarhus Denmark

**Keywords:** mobile phone, app, patient decision aid, dialysis, decisional conflict, usability

## Abstract

**Background:**

The Dialysis Guide (DG) is a patient decision aid (PDA) available as an app and developed for mobile phones for patients with chronic kidney disease facing the decision about dialysis modality.

**Objective:**

The aim of this study was to uncover the applicability of the DG as a PDA.

**Methods:**

The respondents completed a questionnaire before and after using the DG. The respondents' decisional conflicts were examined using the Decisional Conflict Scale, and the usability of the app was examined using the System Usability Scale (SUS). The change in decisional conflict was determined with a paired *t* test.

**Results:**

A total of 22 respondents participated and their mean age was 65.05 years; 20 out of 22 (90%) had attended a patient school for kidney disease, and 13 out of 22 (59%) had participated in a conversation about dialysis choice with a health professional. After using the DG, the respondents' decisional conflicts were reduced, though the reduction was not statistically significant (*P*=.49). The mean SUS score was 66.82 (SD 14.54), corresponding to low usability.

**Conclusions:**

The DG did not significantly reduce decisional conflict, though the results indicate that it helped the respondents decide on dialysis modality. Attending a patient school and having a conversation about dialysis modality choice with a health professional is assumed to have had an impact on the decisional conflict before using the DG. The usability of the DG was not found to be sufficient, which might be caused by the respondents’ average age. Thus, the applicability of the DG cannot be definitively determined.

## Introduction 

This paper sheds light on the applicability of a patient decision aid (PDA), the Dialysis Guide (DG) [1], which is available as an app for mobile phones and is made in accordance with the International Patient Decision Aid Standards (IPDAS) [2]. The DG is for patients with chronic kidney disease facing the choice of dialysis modality. The DG is a further development of a PDA in paper format, currently used at four hospitals in Denmark [3].

According to IPDAS, the purpose of a PDA is to improve the quality of decisions to enable patients to make informed, value-based decisions [4]. PDAs must make the decision explicit, contain information, and clarify advantages and disadvantages. The aim is to create agreement between the decision and individual values and preferences [5]. PDAs contribute to reduction of decisional conflicts and positively affect patients’ basis for making a decision [6]. Some PDAs have been developed for patients with chronic kidney disease; however, studies of the effects of PDAs are few [7]. Thus, it is relevant to measure whether the DG reduces patients’ decisional conflicts and helps them decide.

A systematic literature search was performed in the Cumulative Index of Nursing and Allied Health Literature (CINAHL) and PubMed databases to identify evaluated PDAs in app format; one PDA in app format for iPads was found [8]. Multiple studies regarding the assessment of online PDAs were also found. Grey literature searches in The Ottawa Hospital Research Institute’s database [9] and the Facebook group *Shared@ Shared Decision-Making Network* supported this, and one more PDA in app format for mobile phones was found [10]. Only a few studies about PDAs in app format were found; none of them covered choice of dialysis modality.

Mobile apps have become easily accessible. In 2017, 84% of Danish families had a mobile phone [11]. Thus, it can be questioned why there are so few PDAs in app format. This is supported by the concept *telemedicine*, which, according to the World Health Organization, includes Web-based apps. The aim of telemedicine is to improve health results and provide clinical support through the use of information and communication technology across physical and geographical barriers [12]. The European Commission regards telemedicine as a solution to demographic changes with more elderly patients and, thus, more chronic diseases [13]. It is important to consider the advantages and disadvantages of apps [14]. The app format makes it easy to update PDAs and the material is always easily accessible. However, not all patients will necessarily benefit from PDAs in the app format. Moreover, patients with no access to technology, as well as the elderly with limited technological knowledge, may find apps less beneficial [15]. A PDA for dialysis choice was required by the Renal Association in Denmark; this was a request made by the patients.

The aim of this study was to examine whether the app, the DG, is applicable as a PDA for patients with chronic kidney disease to decide on dialysis modality. The following hypotheses were made: 

Hypothesis 1: The DG reduces the patient’s decisional conflicts.

Hypothesis 2: The DG has a high level of usability.

## Methods

### Study Design 

Initially, we made a qualitative pilot study to adjust the DG. After this, a quantitative study design was used. This study design was developed as a hypothetico-deductive, cross-sectional study where data were collected through a pre- and postintervention questionnaire and used to examine the applicability of the DG. The method for assessing the DG was supported by the available literature [8,16-21]. 

### The Dialysis Guide 

**Figure 1:**
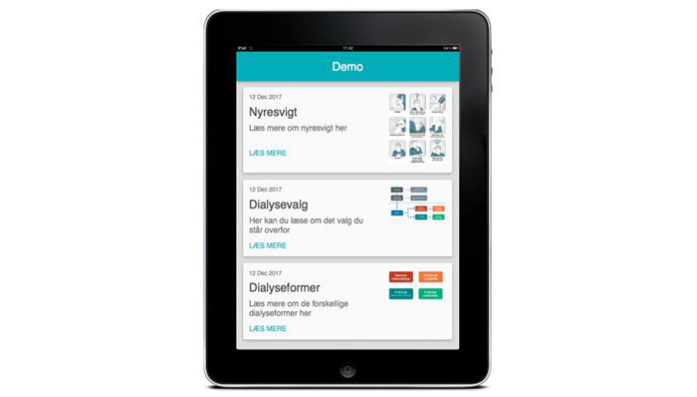
Screenshot of the Dialysis Guide index.

The purpose of the DG is to support patients to clarify their values when choosing dialysis modality. The DG includes information and a test. The information focuses on kidney failure, dialysis choice, and dialysis modalities, and the app has a glossary (see Figure 1). 

**Figure 2:**
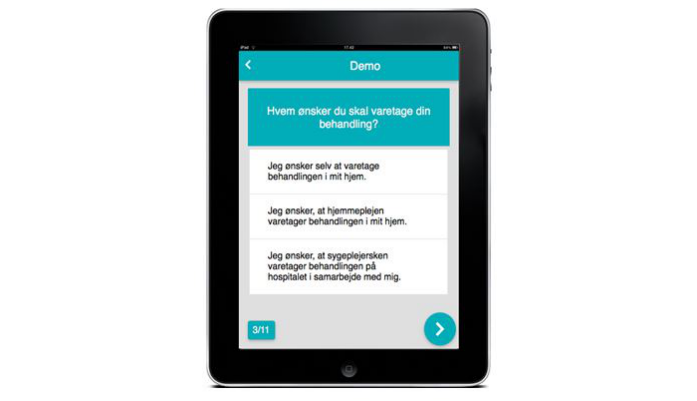
Screenshot of the Dialysis Guide test.

The dialysis modalities to choose from include home hemodialysis, peritoneal dialysis, peritoneal dialysis with help, or hemodialysis at hospital.  The test contains 11 questions to match the advantages and disadvantages of the different dialysis modalities to the patient’s preferences for each dialysis modality (see Figure 2). The individual patient’s test result is presented in the end. 

### Technical Development 

The DG is a responsive front-end Web app developed in HTML5. The front end communicates via http and JavaScript Object Notation (JSON) with a server-based back end, which is based on Drupal content management system (CMS), version 7.38 (Dries Buytaert). This is used to administer all the content published in the front-end app. The back end is executed via a Linux-based (The Linux Foundation) Apache Web server (The Apache Software Foundation) that runs a Hypertext Preprocessor (PHP), version 7.0.26 (The PHP Group), with two attached database servers that run the MySQL (Structured Query Language), version 5.7.20 (Oracle Corporation) [22].

### Pilot Study

The pilot study was performed as two focus groups aimed at acquiring new knowledge [23]. These took place in September and October 2017 in two Danish regions; 6 and 11 patients participated, respectively. They were conducted by the second author in accordance with Kvale and Brinkmann [24]. Data analysis was conducted by all authors using systematic text condensation [25].

We found that the informants’ ages and the severities of the disease were significant for a patient’s approach to the DG. Some experienced that the DG was introduced too early in their disease trajectory. Multiple informants stated that apps were not for the elderly. Furthermore, several participants expressed a lack of time to try out the app. The DG information was found relevant and easy to understand. Constructive feedback in relation to the wording of some questions in the test was also given. In addition, a regional difference in the motivation for the use of the app was identified. The findings were used to adjust the DG as well as to construct the demographic questions for the evaluation of the app.

### Selection of Respondents 

People aged 18 years or older with chronic kidney disease and an estimated glomerular filtration rate (eGFR) between 10 and 20 mL/min/1.73 m^2^ were included in the study. Patients unable to read and understand Danish, who did not use electronic devices, or with cognitive deficits were excluded. Likewise, patients who had previously received dialysis or who had already started dialysis were also excluded. Respondents were recruited by nurses from eight of 14 renal departments in Denmark between November 23, 2017, and June 30, 2018. The nurses informed the patients about the study and obtained patients’ email addresses. The questionnaires were subsequently forwarded to the patients by email, including a link to the DG.

### Decisional Conflict 

To clarify whether the DG reduced patients’ decisional conflicts, the validated 16-statement Decisional Conflict Scale (DCS) was used. The scale uses a 5-point Likert scale from 0 (*Strongly agree*) to 4 (*Strongly disagree*) [26,27]. The scale measures whether the PDA facilitates effective decision making as well as insecurities in relation to decision making [28]. SurveyXact (Rambøll) [29] was used for data collection. The preintervention questionnaire contained demographic questions (ie, age, gender, region, participation in a conversation regarding dialysis choice, and participation in a patient school) and measured the respondents’ decisional conflicts using the DCS (DCS1). The respondents were recommended to use the DG for 20 minutes before they completed the postintervention questionnaire. Then, the respondents’ decisional conflicts were measured again using the DCS (DSC2). The data analysis was performed using Excel (Microsoft Corporation) [30]. The means and SDs were calculated for the DCS. The results for DCS1 and DCS2 were compared using a paired *t* test. Data obtained from the DCS were rated using a total score between 0 and 100. In the DCS, a score of 0 is considered as no decisional conflict, a score of 100 is considered as an extremely high degree of decisional conflict, and a score lower than 25 is associated with making a decision. The subscores—*Uncertainty*, *Information*, *Values Clarity*, *Support*, and *Effective Decision*—were calculated [31].

### Usability

To assess the usability of the DG, the System Usability Scale (SUS) was used. The SUS consists of 10 questions that also use a 5-point Likert scale from 1 (*Strongly disagree*) to 5 (*Strongly agree*) [32]. The scale was chosen as it was validated and developed to assess the usability of software [33].

Data were collected postintervention. Data obtained from the SUS were rated using a total score between 0 and 100, and means and SDs were calculated. In the SUS, a score of 0 is considered a low degree of usability and a score of 100 as a high degree of usability. A score above 68 is higher than the average SUS score and a score of 76 shows good usability [33]. The individual questions in the SUS were presented with the percentage of answers spread between the different choices on the Likert scale.

### Ethical Considerations

The Central Denmark Region Committee on Health Research Ethics [34] and The Danish Data Protection Agency [35] have approved this study. All involved patients have given informed consent. Data were anonymized, safely stored, and shredded after use.

## Results

### Participant Flow and Characteristics

A total of 33 respondents were recruited; 28 (85%) of those completed the preintervention questionnaire and 22 (67%) also completed the postintervention questionnaire. Only the respondents who completed both questionnaires were included (22/33, 67%) (see Table 1). The mean age of the respondents was 65.06 years; 15 respondents were men (68%) and 7 were women (32%). Respondents from three out of five regions in Denmark were represented. A total of 20 out of 22 respondents (91%) had already attended a patient school, while 13 out of 22 (59%) had participated in a conversation about dialysis choice. Three electronic devices were used in the study: 12 out of 22 participants (55%) used a mobile phone, 5 out of 22 (23%) used an iPad or tablet, and 5 out of 22 (23%) used a computer.

**Table 1:** Demographic characteristics (N=22).

Characteristics		Value
Age (years), mean (range)		65.05 (44-86)
**Gender, n (%)**		
	Female	7 (32)
	Male	15 (68)
**Region, n (%)**		
	North Denmark Region	0 (0)
	Central Denmark Region	10 (45)
	Region of Southern Denmark	0 (0)
	Region Zealand	1 (5)
	Capital Region of Denmark	11 (50)
**Attended patient school, n (%)**		
	Yes	20 (91)
	No	2 (9)
**Attended a conversation about dialysis with a health practitioner, n (%)**		
	Yes	13 (59)
	No	9 (41)
**Electronic device used, n (%)**		
	Mobile phone	12 (55)
	iPad or tablet	5 (23)
	Computer	5 (23)

### Decisional Conflict

A comparison of the results for DCS1 and DCS2 showed a difference in mean values and SDs (see Table 2). For DCS1, the total mean decisional conflict was 26.42 (SD 18.12) and for DCS2 the mean was 25.21 (SD 16.93). Thus, no significantly reduced decisional conflict was found (paired *t* test: difference=-1.21, *P*=.49). The remaining subscores were not significant either. The respondents’ DCS1 scores for *Information* and *Support* were lower than 25, which was also the case for DCS2. The *Uncertainty* subscore was reduced the most with a fall of 3.41. Both the *Values Clarity* subscore and the *Support* subscore were higher after use of the DG.

The total mean for decisional conflicts was lower for respondents who had attended a conversation about dialysis choice before using the DG, while it was only reduced for those who had not attended one (see Table 3). The women’s mean decisional conflict was lower compared to that of the men. The women’s mean decisional conflict rose after having used the DG, whereas it was reduced among the men.

**Table 2:** Changes from preintervention Decisional Conflict Scale (DCS1) to postintervention Decisional Conflict Scale (DCS2) (N=22).

Score	DCS1, mean (SD)	DCS2, mean (SD)	Difference (paired *t* test)	*P* value
Total score	26.42 (18.12)	25.21 (16.93)	–1.21	.49
*Uncertainty* subscore	30.30 (24.87)	26.89 (19.57)	–3.41	.13
*Informed* subscore	22.73 (14.13)	21.97 (15.97)	–0.76	.67
*Values Clarity* subscore	25.38 (18.81)	28.03 (18.64)	2.65	.41
*Support* subscore	21.14 (17.45)	21.97 (18.10)	0.83	.83
*Effective Decision* subscore	29.26 (21.42)	26.70 (19.88)	–2.56	.29

**Table 3:** The meaning of demography for the Decisional Conflict Scale.

Demographic	DCS1^a^ total score, mean (SD)	DCS2^b^ total score, mean (SD)
Attended a conversation about dialysis with a health practitioner	20.67 (9.38)	21.75 (9.85)
Did not attend a conversation about dialysis with a health practitioner	34.72 (24.47)	31.60 (22.72)
Male	29.02 (15.91)	27.15 (13.71)
Female	20.76 (22.48)	20.98 (23.14)

^a^DCS1: preintervention Decisional Conflict Scale.

^b^DCS2: postintervention Decisional Conflict Scale.

### Usability

The majority of the answers to the individual questions in the SUS ranged from 2 to 4 on the Likert scale. The mean for the overall SUS score was 66.82 (SD 14.54). The average SUS scores for the individual devices were 69.58 for mobile phones, 66.00 for iPads or tablets, and 61.00 for computers. See Table 4 for a summary of SUS response scores for each question.

**Table 4:** System Usability Scale (SUS) answers (N=22).

Question	Response, n (%)				
	Strongly disagree	Disagree	Neither agree nor disagree	Agree	Strongly agree
I think that I would like to use the Dialysis Guide frequently	1 (5)	0 (0)	7 (32)	11 (50)	3 (14)
I found the Dialysis Guide unnecessarily complex	2 (9)	7 (32)	11 (50)	2 (9)	0 (0)
I thought the Dialysis Guide was easy to use	7 (32)	3 (14)	8 (36)	4 (18)	0 (0)
I think that I would need the support of a technical person to use the Dialysis Guide	1 (5)	0 (0)	5 (23)	14 (64)	2 (9)
I found the various functions in the Dialysis Guide were well integrated	2 (9)	10 (45)	8 (36)	1 (5)	1 (5)
I thought there was too much inconsistency in the Dialysis Guide	2 (9)	10 (45)	8 (36)	1 (5)	1 (5)
I would imagine that most people would learn to use the Dialysis Guide very quickly	0 (0)	2 (9)	4 (18)	13 (59)	3 (14)
I found the Dialysis Guide very cumbersome to use	5 (23)	10 (45)	7 (32)	0 (0)	0 (0)
I felt very confident using the Dialysis Guide	0 (0)	1 (5)	7 (32)	8 (36)	6 (27)
I needed to learn a lot of things before I could get going with the Dialysis Guide	4 (18)	5 (23)	8 (36)	5 (23)	0 (0)

## Discussion

The reduction of the respondents’ decisional conflicts as a result of using the DG was not significant. Thus, it is difficult to determine the extent to which the DG can help patients make a decision regarding the choice of dialysis modality. Therefore, it cannot be concluded that the DG meets the purpose of PDAs described by IPDAS, which is to improve the quality of decisions to facilitate informed and value-based decisions [3].

The usability of the DG is problematic because of the low number of respondents and their different answers in the SUS, with scores mainly around the middle of the Likert scale. Therefore, it cannot be confirmed that the DG has a high degree of usability. Likewise, it was difficult to shed light on the applicability of the app format for mobile phones, as all three types of devices were represented.

The results for the subscores in the DCS were not significant either, which might be due to the low number of respondents. However, the results indicated that the respondents received sufficient information, experienced an improved decision quality, and were less uncertain about their decision. In the evaluation of a similar PDA in paper format, the patients obtained an increased knowledge of the different dialysis modalities and became more prepared to make a decision [36]. This is in line with our study, as indications suggest that the DG improved respondents’ levels of information as well as reduced their uncertainty. 

The *Information* and *Support* subscores were below 25 in DCS1, which indicate that the patients had already received sufficient information and aid in making their decisions about dialysis modality before use of the DG [27]. Therefore, it is doubtful that these subscores could be reduced further through the use of the DG.  However, a minor reduction of the *Information* subscore was seen. Already prior to using the DG, the respondents’ decisional conflicts neared 25. This can be explained by the fact that 91% had participated in a patient school before using the DG. As the *Information* subscore in DCS1 was low, it is assumed that the respondents had received adequate information at the patient schools to make a decision. In other studies, it was also found that patients with chronic kidney disease who had already been educated on the subject had a lower degree of decisional conflict than patients who had not received any information [37,38]. One of the studies supports the notion that information at a patient school may have an impact on the applicability of a PDA. Whether or not patients had been exposed to a PDA—beyond being exposed to information—we found that the DG did not have an impact on patients’ decisional conflicts. It was also found that decisional conflict only appeared among those respondents who had not yet had a conversation with a professional about dialysis choice [38]. This could indicate that the DG is mainly applicable for patients faced with the choice of dialysis modality, but who had not yet received information at a patient school or discussed dialysis choice with a professional. 

Prior to use of the DG, the female respondents had a decisional conflict below 25. This indicates that they had already decided on a type of dialysis at this point [27]. On the other hand, the men had not decided on their choice before use of the DG, but their decisional conflict was reduced after its use. This indicates that the DG helped the men become more confident in making a decision. However, another study on significant factors for decisional conflict when choosing dialysis modality did not find that gender was a factor regarding decisional conflict [37]. 

The mean for the total SUS score was below the average SUS score, but the mean value when using a mobile phone was above this average score. Usability is considered highest when using a mobile phone, which is in contrast to a study evaluating a PDA for an iPad. Here, usability was higher on an iPad, as the size of the screen was bigger [8]. So far, there are only few PDAs in app format for mobile phones. However, examples of patients finding PDAs for mobile phones usable and easy to navigate exist [10]. It is also presumed that a high degree of usability can be achieved for PDAs for mobile phones. However, the patients who used these had extensive knowledge of mobile phones, which might have impacted on the high usability [39,40]. This could also be the case in this study, as the respondents who chose to use a mobile phone would most likely choose it because they were familiar with this device. 

The mean age for the respondents was 65.05 years, which is similar to the patient population’s mean age in the Danish Nephrology Registry’s Annual Report, 2016 [41]. It is presumed that the mean age may have an impact on the assessed usability of the DG, as the cognitive and physical abilities of older and younger participants differ. This may influence the older participants’ use of Web-based telemedicine solutions [42]. In another study, researchers found that the usability of an online PDA was higher for patients under the age of 36 years [19]. 

The limitations of this study make it difficult to assess the applicability of the DG. The method of distribution entailed a weakness as it was not possible to make a dropout analysis. The number of respondents and the low response rate was also a weakness. The reason for the low number of respondents might be the lack of recruitment from some of the invited renal departments. Doubts about the validity of some of the email addresses might have impacted on the response rate. In other studies evaluating the use of PDAs in the same patient group, a response rate of 70% or lower was also seen [38,43]. One of these studies described that the nurses’ recruitment of patients might be important to the outcome [36]. The nurses’ approach to, and presentation of, the PDA may also have an impact on patients’ views of the applicability [10]. Despite the inclusion and exclusion criteria, the nurses’ subjective recruitment might have impacted on the results. The low number of respondents and low recruitment could mean that not all Danish regions were represented; therefore, it was not possible to assess whether the geographical location had an impact on the respondents’ answers. Despite the recommended time for using the DG, not knowing how long the individual respondents used the app was a limitation.

On the basis of this study, it cannot be definitively concluded that the DG is applicable as a PDA for patients with chronic kidney disease deciding on dialysis modality. The DG did not reduce respondents’ decisional conflicts significantly. Hence, Hypothesis 1 cannot be confirmed. However, a reduction of respondents’ decisional conflicts was seen after use of the DG, but indications suggest that this is limited if the patient had received information at a patient school or attended a conversation about dialysis choice with a professional beforehand. 

It can be concluded that the usability of the DG is not sufficiently clarified at present, which means that Hypothesis 2 is disproved. The low usability might be a result of the respondents’ mean age. However, the usability was assessed as higher when using a mobile phone.

The limited knowledge about PDAs in app format and the number of respondents in this study mean that there is a need for further research to determine the applicability of the DG. It might be relevant to examine the difference between the applicability of PDAs in paper and app formats in a randomized controlled trial. If the DG is to be used in shared decision making, it is also relevant to examine the health professional’s view of the applicability of the DG.

## Abbreviations

CINAHL: Cumulative Index of Nursing and Allied Health Literature
CMS: content management system
DCS: Decisional Conflict Scale
DCS1: preintervention Decisional Conflict Scale
DCS2: postintervention Decisional Conflict Scale
DG: Dialysis Guide
eGFR: estimated glomerular filtration rate
IPDAS: International Patient Decision Aid Standard
JSON: JavaScript Object Notation
PDA: patient decision aid
PHP: Hypertext Preprocessor
SQL: Structured Query Language
SUS: System Usability Scale
